# Oral manifestations in patients with chronic hepatitis C

**DOI:** 10.1186/s12903-025-06307-5

**Published:** 2025-06-09

**Authors:** Michał Brzdęk, Joanna Gałuszka-Garnuszek, Krystyna Dobrowolska, Kinga Brzdęk, Jakub Janczura, Olga Tronina, Magdalena Kal, Piotr Stępień, Dorota Zarębska-Michaluk

**Affiliations:** 1 Collegium Medicum, Jan Kochanowski University, Kielce, Poland; 2 Department of Gastroenterology, Medical University of Lodz, Lodz, Poland; 3 Institute of Literary Studies and Linguistics, Jan Kochanowski University, Kielce, Poland; 4 Department of Transplant Medicine, Immunology, Nephrology and Internal Diseases, Medical University of Warsaw, Warsaw, Poland; 5 Ophthalmic Clinic of the Voivodeship Hospital in Kielce, Kielce, Poland; 6 Department of Infectious Diseases and Allergology, Jan Kochanowski University, Kielce, Poland

**Keywords:** Hepatitis C virus, Chronic hepatitis C, Extrahepatic manifestations, Oral pathologies, Oral lichen planus

## Abstract

**Background:**

Hepatitis C virus (HCV) infection, a systemic disease characterized by extrahepatic manifestations (EMs), affects approximately 50 million people worldwide. Recognizing EMs, which may involve multiple organs and systems, is crucial for timely diagnosis and effective antiviral therapy.

Purpose of the study was to investigate extrahepatic symptoms occurring in the oral mucosa in HCV-infected patients.

**Methods:**

The observational study included 153 consecutive patients with chronic hepatitis C and healthy controls. Data collection encompassed demographic parameters, medical history, laboratory results, and oral examinations, which included evaluation of dry mouth, pain and burning in the mouth and on the tongue, pain in the angles of the mouth, bad breath, gingival bleeding, dysphagia and taste disorders using scales designed for this purpose, clinical and dental examination.

**Results:**

Subjective oral symptoms were twice as common in the study group as in controls with the most frequent dry mouth, followed by oral pain, and burning in the mouth. Pathological changes (oral candidiasis, angular cheilitis and lichen planus), were identified in 73.2% of patients, compared to 32% in the control group. Oral hygiene was worse in the study group with a median score of 1.8 compared to 1.1 as assessed by the Oral Hygiene Index scale. The incidence of Mikulicz's aphthae, papillomas, fibromas and sublingual varices did not reach statistically significant differences. The study group had fewer teeth with dental fillings. Additionally, age ≥ 40 years and GT1 infection were identified as independent predictors of oral pathologies in HCV-infected patients.

**Conclusions:**

In patients with chronic HCV infection, oral mucosal pathologies were significantly more common compared to controls, with candidiasis, angular cheilitis, and oral lichen planus being the most frequently observed conditions. Subjective symptoms such as dry mouth, oral pain, and burning were also markedly higher in the HCV group. Age ≥ 40 years and GT1b HCV genotype were identified as independent positive predictors of oral mucosal lesions.

## Introduction

Hepatitis C virus (HCV) is an enveloped, positive-strand RNA virus belonging to the genus Hepacivirus in the family Flaviviridae [1]. It is a hepatotropic virus, meaning that it infects liver cells, which are its main target in the patient's body [2]. As a result of infection of hepatocytes, necroinflammatory changes develop in the liver, clinically manifested as hepatitis, which may progress to cirrhosis with the risk of decompensation and hepatocellular carcinoma (HCC) [1]. These conditions are the most serious complications of HCV infection, affecting about one-fifth of patients out of an estimated by World Health Organization (WHO) 50 million infected worldwide [3]. However, it is a well established that HCV has tropism not only for liver cells, and the symptoms and conditions experienced by patients are not limited to this organ which makes chronic hepatitis C (CHC) a systemic disease [4]. The basis of multiple extrahepatic manifestations (EMs) of HCV is the lymphotropism of the virus, the ability to replicate in non-hepatic tissues, the complex interactions it enters into with the host immune system, and the possibility of triggering autoimmune reactions [5]. Symptoms beyond the liver can affect practically any organ and system, the first to be described is mixed cryoglobulinemia, which occurs in about one-third of patients with CHC, in most of them asymptomatic in nature, and its pathogenetic link to HCV is documented and strong [6]. If cryoglobulinemia is clinically evident, the most typical symptoms include vascular purpura, weakness, bilateral arthralgia without joint destruction, peripheral polyneuropathy, and glomerulonephritis. Much rarer than cryoglobulinemia, which is the most common EM, but equally strongly associated with HCV infection pathogenetically are non-Hodgkin's B-cell lymphomas [4]. Extrahepatic symptoms of the hematopoietic system also include thrombocytopenia, which occurs either due to direct cytotoxicity of the HCV, which can replicate in megakaryocytes, or indirectly through immunological mechanisms [7]. Thyroid abnormalities, including clinically overt autoimmune thyroiditis and isolated increases in antithyroid antibody levels, are probably mediated by a complex mechanism involving helper T cells and are the most common endocrine manifestations [4]. The association of chronic HCV infection with metabolic diseases, such as insulin resistance and type 2 diabetes, as well as with an increased risk of cardiovascular diseases has also been documented [8, 9]. The most spectacular EMs, because visible to the naked eye, are manifestations involving the skin, such as lichen planus, porphyria cutanea tarda, and vasculitis with purpura [5]. Among those that make the daily functioning of patients with CHC particularly difficult are problems with memory and concentration, depression, fatigue, and cognitive disorders, which are believed to be immune-mediated, but a direct effect of cytotoxic HCV on the nervous system is also suspected [5].

The disorders mentioned above do not exhaust the list of all conditions with the relationship with chronic HCV infection has been confirmed or suspected, they may affect many systems outside the liver, additionally burdening the patient with comorbidities that worsen the prognosis and increase non-liver-related mortality [5]. Conversely, the occurrence of extrahepatic manifestation, and according to available data up to two-thirds of CHC patients experience at least one, may be the first visible sign of infection, which usually remains undiagnosed for many years due to the absence of hepatic symptoms [4].

Therefore, awareness of HCV-related EMs among non-hepatology specialists is so important. It enables the initiation of diagnostics, which results in the diagnosis of the infection and thus the implementation of antiviral therapy. In the era of safe and highly effective direct-acting antivirals (DAA), HCV eradication gives such patients a chance not only to improve their liver condition but also to reduce the incidence of extrahepatic symptoms [4].

This study addresses the need for updated data on HCV-related extrahepatic symptoms in the DAA era, as understanding these manifestations is essential for early identification of HCV infection by physicians across various specialties. The aim of this study was to assess the prevalence and types of oral mucosal symptoms and pathologies in patients with chronic hepatitis C (HCV), in comparison to a matched control group. In addition, we aimed to identify clinical and demographic predictors of oral manifestations in this population. By highlighting the characteristic oral findings associated with HCV infection, we also seek to raise awareness among dental professionals and emphasize the potential role of dentists in the early identification and referral of patients for HCV diagnosis and antiviral treatment.

## Materials and Methods

### Study population and data collection

The study included consecutive patients with CHC who were diagnosed in the Department of Infectious Diseases of the Provincial Hospital in Kielce between January 1, 2018, and December 31, 2018 (study group) in line with national recommendations [10]. According to patient declarations in the medical history, none of the patients included in the study had a history of alcohol abuse or nicotine addiction. In terms of smoking status, patients were classified either as"Never smokers"(defined as adults who have never smoked, or who have smoked fewer than 100 cigarettes in their lifetime) or"Someday smokers"(defined as adults who have smoked at least 100 cigarettes in their lifetime and currently smoke, but not on a daily basis), according to the CDC definitions [11]. Regarding alcohol consumption, patients reported intake within the limits of the national low-risk drinking recommendations for Poland, published by the European Commission, i.e., up to 40 g of alcohol per day for men and up to 20 g for women, consumed on no more than five days per week [12]. All patients in the study population were characterized by good socioeconomic conditions. The control group included healthy individuals with negative result for the presence of the anti-HCV antibodies who were family members and employees of the Military Specialist Medical Clinic in Kielce. Healthy subjects with no chronic diseases, including diabetes, autoimmune disorders or malignancies, were recruited for the control group. None of the individuals in the control group were chronically using medications known to affect the oral mucosa, such as nonsteroidal anti-inflammatory drugs, antihypertensive drugs, antimalarials, immunosuppressants, antidepressants or other agents known to cause lichenoid or xerostomic reactions.

The following demographic parameters were collected: gender, age, and body mass index (BMI). Oral and dental examinations in both groups were performed at the Military Specialist Medical Clinic in Kielce. The analysis used data from patients'medical records, including laboratory, virological and clinical parameters.

### Subjective dental examination of the oral cavity

A medical history of subjective oral complaints was taken including an assessment for dry mouth, pain and burning in the oral cavity, pain and burning of the tongue, pain at the corners of the mouth, unpleasant breath odor, gum bleeding, dysphagia and taste disturbances. Pain intensity was assessed using the Numeric Rating Scale (NRS), where patients were asked to rate their pain verbally on a scale from 0 (no pain) to 10 (worst imaginable pain). The responses were recorded directly in the patients'medical documentation. Although NRS is a general pain scale, its applicability to orofacial pain assessment is supported in the literature [13]. The mouth dryness severity was assessed using the Challacombe Scale. The scale is based on ten symptoms associated with saliva deficiency in the mouth [14]. Each of the visual signs of dryness was given one point and the total score formed the basis for interpreting the results.

- 1. Adhesion of the mirror to the oral mucosa.
- 2. Adhesion of the mirror to the mucous membrane of the tongue.
- 3. Presence of foamy saliva in the mouth.
- 4. Insufficiency of saliva on the floor of the mouth.
- 5. Smoothing of the tongue with reduction of papillae on the tongue.
- 6. Shiny and smooth surface of the gums.
- 7. Glassy mucosal surface on the palate.
- 8. Lamellar tongue.
- 9. Cervical caries on more than two teeth.
- 10. Deposit on teeth and palate.

The scores obtained allowed interpretation of the results by dividing the level of subjective perception of mouth dryness into no dryness, moderate, medium and severe dryness [14].

### Clinical examination of the oral mucosa

In both groups, the visual assessment of the oral mucosa was performed under artificial light to identify pathological changes. The assessment included the presence of lesions such as lichen planus, Mikulicz’s aphthae, angular cheilitis, papilloma, fibroma, candidiasis and sublingual varices. Diagnoses of oral mucosal diseases were made based on an analysis of clinical symptoms, the patient's medical history, and physical examination. In clinically questionable cases, a biopsy was performed for histopathological evaluation. The biopsies were analyzed at the Non-Public Health Care Facility Department of Pathology in Kielce.

### Dental physical examination of the oral cavity using indicators

Assessment of dental status was performed under artificial light using a diagnostic kit according to the WHO criteria [15]. The following clinical indicators were collected:

- The Decayed-Missing-Filled (DMF) Index used to assess caries incidence. The DMF index is the sum of teeth affected by caries on one or more surfaces, teeth lost due to caries and teeth with dental fillings on one or more surfaces [16].
- Simplified Oral Hygiene Index (OHI-S) for the assessment of oral hygiene, taking into account the presence of plaque and tartar on the buccal surfaces of teeth 16, 11, 26, 31 and on the lingual surfaces of teeth 36, 46 [17].

In the absence of an indicator tooth, the examination included the adjacent tooth. Missing teeth constituted an exclusion from the oral hygiene assessment examination. Based on the above indicator, four levels of oral hygiene were distinguished- very good (values 0.0–0.0), good (0.1–1.2), sufficient (1.3–3.0) and insufficient (3.1–6.0).

- Community Periodontal Index of Treatment Needs (CPITN) to assess periodontal status [18, 19]. The depth of the gingival pockets between the teeth and the periodontal tissues was examined using a 621 WHO probe with a 0.5 mm ball end and a mirror. The index teeth were: 11, 31, 17, 16, 26, 27, 36, 37, 46, 47. To our analysis, we used the worst periodontal condition. Teeth fitted with prosthetic crowns, sextants with fewer than 2 teeth and patients whose dental condition did not allow the above indicator to be determined were excluded from the study.

Based on the CIPTN index, patients were categorized into three groups of periodontal treatment needs.

### Microscopic evaluation for Candida albicans

Material was collected from both control and study group subjects with a swab spatula from the dorsal surface of the tongue and cheek, and then transferred to a basic slide and mixed with a 5% potassium hydroxide (KOH) solution. Microscopic evaluation of the preparation included the assessment of the presence of blastopores, pseudohyphae, and true hyphae.

### Characteristics of patients with HCV infection

In the group of patients with CHC the following clinical parameters were gathered: the presence of comorbidities and concomitant medications, the history of previous antiviral therapy (including interferon-based regimens such as PegIFN + RBV, or other therapies) and the presence of EM of HCV infection unrelated to the oral mucosa.

Laboratory tests in the HCV-infected group were performed at the Laboratory of the Provincial Hospital in Kielce. Complete blood count included leukocyte (reference range: 4.1–10.9 × 10⁹/L), erythrocyte (4.2–6.0 × 10^12^/L), hemoglobin (12.0–18.0 g/dL), and platelet (140–440 × 10⁹/L) counts. Liver function parameters, including ALT and AST (reference range for both: 5–50 IU/L), bilirubin (0.1–1.3 mg/dL), and albumin levels, were measured using standard kinetic and colorimetric methods on the AU 400 Olympus analyzer. Anti-HCV antibodies, HBsAg, and anti-HIV antibodies were assessed using third- or fourth-generation ELISA or microELISA assays. Cryoglobulins were identified in serum based on their ability to reversibly precipitate after incubation at + 4 °C for 4–6 days. Quantitative HCV RNA levels and genotype determination were performed using GeneProof Real-Time PCR (Roche Lightcycler 2.0) in the Molecular Biology Unit of the same hospital, with a method sensitivity of 10 IU/L.

Elastography to assess liver fibrosis was performed with the AIXPLORER. Results were interpreted in accordance with the 2018 recommendations of the European Association for the Study of Liver Diseases (EASL) using a METAVIR scale [20].

### Ethical considerations

This observational study was conducted after the approval of the Bioethics Committee of the Jan Kochanowski University in Kielce by resolution No. 18/2018 of 26.03.2018, and access to medical records and use of patient treatment data was made with the prior consent of the Hospital Management. All patients were included in the study after providing all information regarding the purpose of the study and obtaining their written consent to participate in the study.

### Statistical analysis

Analysis of qualitative variables was performed by calculating the number and percentage. Comparisons of these variables across groups were made using the chi-square test or Fisher's exact test. Analysis of quantitative variables was performed by calculating the median, quartiles, minimum and maximum. Comparisons between quantitative variables in two groups due to their non-Gaussian distribution, were made using the Mann–Whitney test. Normality of distribution was checked with the Shapiro–Wilk test. Multivariate analysis of the independent effect of multiple variables on a dichotomous variable was performed using multiple logistic regression. Results were presented as OR (odds ratios) coefficients with 95 percent confidence intervals. *P* values of < 0.05 were considered to be statistically significant. Statistical analyses were performed using Statistica v. 13 (StatSoft, Tulsa, OK, United States) and GraphPad Prism 5.1 (GraphPad Software, Inc., La Jolla, CA).

## Results

### Characteristics of the study and control groups

**Fig. 1:**
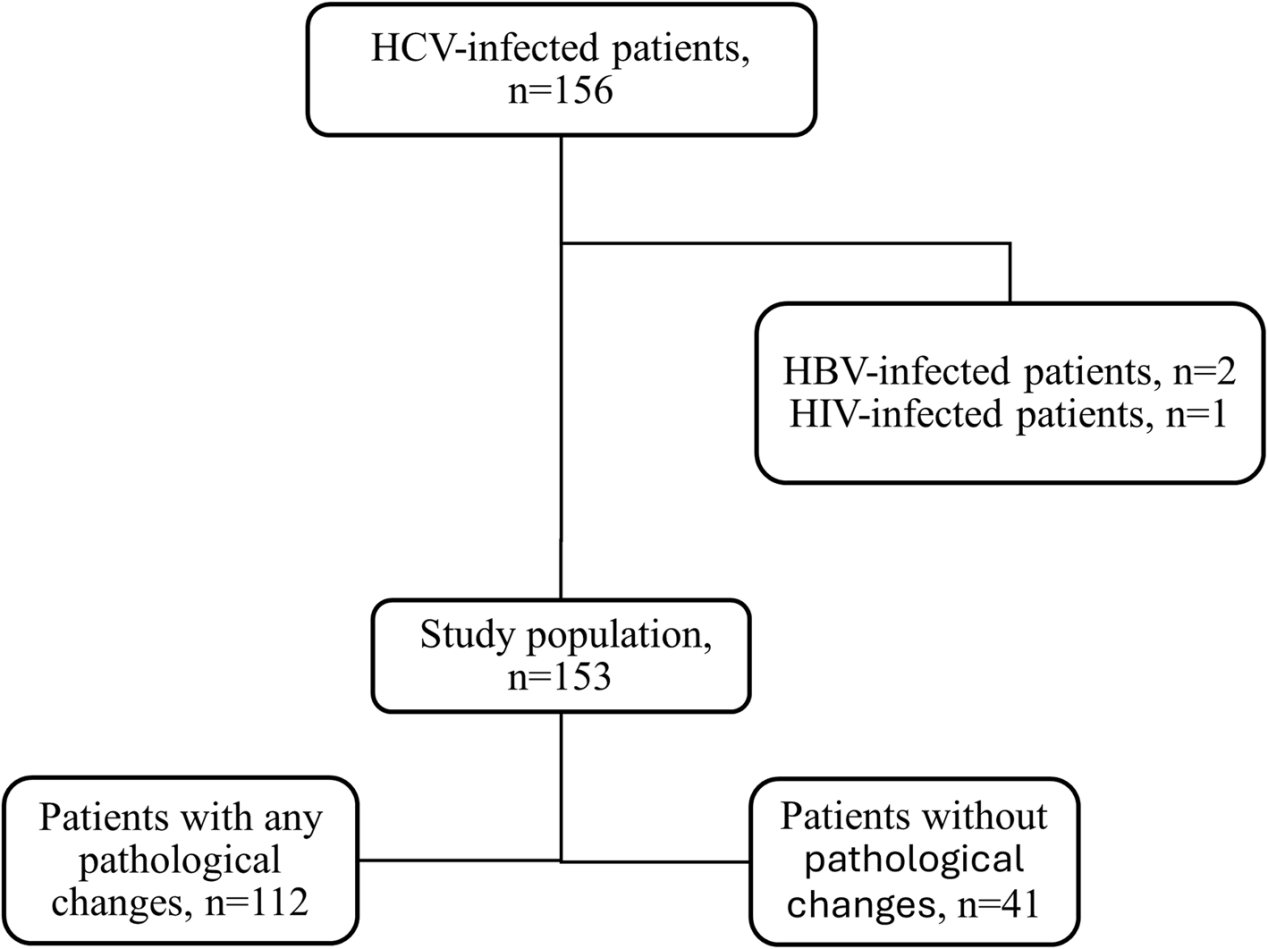
Flow chart of patient selection for the studied population. HBV: Hepatitis B virus; HCV: Hepatitis C virus; HIV: Human immunodeficiency virus

The study included 156 consecutive patients with CHC under the care of the Department of the Infectious Diseases of the Provincial Hospital in Kielce, 3 of whom were excluded from the analysis due to HBV and/or HIV co-infection. The control group consisted of 50 generally healthy individuals without anti-HCV antibodies, who were employees of the Military Specialized Medical Clinic in Kielce or members of their families (Fig. 1).

The median age (Q1-Q3) in the study group was 43 years (33–62) as compared to 50 years (32–58) in the control population (Table 1). In both groups, women predominated, accounting for approximately 60% of the participants. No statistically significant differences between the groups in terms of demographic characteristics were found.

**Table 1:** Characteristics of patients in the study and control groups

Parameter	Study population, *n* = 153	Control group, *n* = 50	*p*
Age (years), median (Q1-Q3)			
All	43 (33–62)	50 (32–58)	0.4463
Females	49.5 (33.5–67.5)	50 (38–58)	0.6997
Males	40 (33–60)	50 (30–54)	0.4345
Gender, n (%)			0.7898
Females	92 (60.1)	29 (58.0)	
Males	61 (39.9)	21 (42.0)	
BMI [kg/m^2^], median (Q1-Q3)	24.91 (22.41–27.89)	24 (22–27)	0.1935
DMF, median (Q1-Q3)	18 (11–26)	17 (11–20)	0.1778
Teeth affected by caries, median (Q1-Q3)	0 (0–2)	0 (0–0)	0.0003
Teeth lost due to caries, median (Q1-Q3)	6 (2–18)	3.5 (1–8)	0.0232
Teeth with dental filling, median (Q1-Q3)	7 (1–10)	10 (8–13)	< 0.0001
OHI-S, median (Q1-Q3)	1.8 (1.15–2.95)	1.1 (0–1.6)	< 0.0001
CPITN, n (%)			< 0.0001
0	9 (5.9)	23 (46.0)	
1	18 (11.8)	11 (22.0)	
2	29 (18.9)	13 (26.0)	
3	51 (33.3)	3 (6.0)	
4	13 (8.5)	0	
No data	33 (21.6)	0	
The need for periodontal treatment, n (%)			< 0.0001
Group 1	27 (17.6)	34 (68.0)	
Group 2	80 (52.3)	16 (32.0)	
Group 3	13 (8.5)	0	
No data^a^	33 (21.6)	0	

^a^Individuals excluded from the study due to the absence of index teeth

Group 1: Patients requiring: Oral hygiene instruction

Group 2: Patients requiring: Oral hygiene instruction, scaling, removal of overhanging fillings

Group 3: Patients requiring: Oral hygiene instruction, scaling, removal of overhanging fillings. Comprehensive treatment

*BMI* Body mass index, *DMF* The Decayed-Missing-Filled, *CPITN* Community Periodontal Index of Treatment Need, *OHI-S* Simplifed Oral Hygiene Index

### Subjective oral symptoms in the study and control groups

**Fig. 2:**
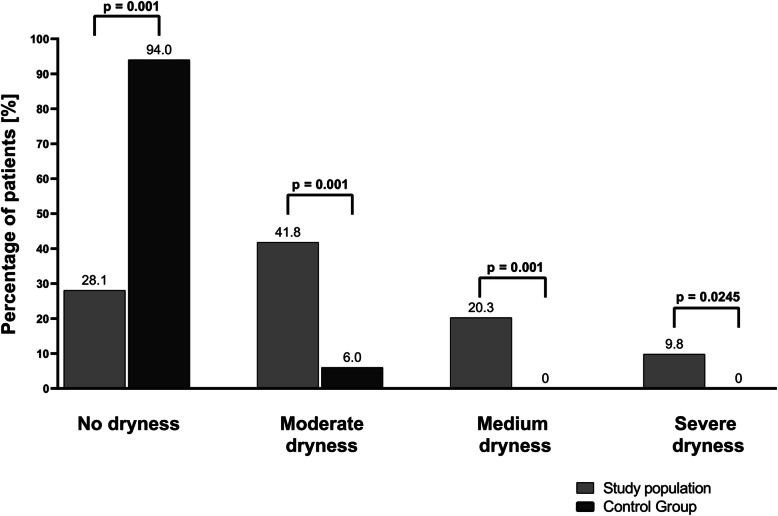
Comparison of mouth dryness severity according to the Challacombe scale in the study and control groups

Subjective symptoms occurred in the study group twice as frequently as in the control group (*p *= 0.0001) (Table 2). The most commonly reported oral symptom among patients in the study group was mouth dryness, affecting 47.1% of individuals. Additionally, dry mouth, pain and burning sensations in the oral cavity, and gum bleeding appeared significantly more often in the study group compared to the control group. According to the Challacombe scale, one-third of the patients in the HCV group experienced medium or severe dryness, in contrast to 0% in the control group (Fig. 2). Moreover, we found that the intensity of oral pain was significantly higher in the study group (*p* < 0.0001) (Table 2).

**Table 2:** Subjective oral symptoms in the study and control groups

Parameter	Study population, *n* = 153	Control group, *n* = 50	p
Prevalence of specific subjective oral symptoms, n(%)			
Any subjective oral symptom	99 (64.7)	16 (32)	0.0001
Mouth dryness	72 (47.1)	3 (6.0)	< 0.0001
Pain and burning in the oral cavity	36 (23.5)	4 (8.0)	0.0147
Pain and burning of the tongue	19 (12.4)	2 (4.0)	0.1113
Pain at the corners of the mouth	8 (5.2)	2 (4.0)	< 0.9999
Unpleasant breath odor	10 (6.5)	1 (2.0)	0.2997
Gum bleeding	10 (6.5)	8 (16.0)	0.0410
Taste disturbances	7 (4.6)	0 (0.0)	0.1972
Dysphagia	7 (4.6)	0 (0.0)	0.1972
Intensity of oral pain, median (Q1-Q3)	2 (0–6)	0 (0–0)	< 0.0001

### Pathological changes the oral mucosa in the study and control groups

**Fig. 3:**
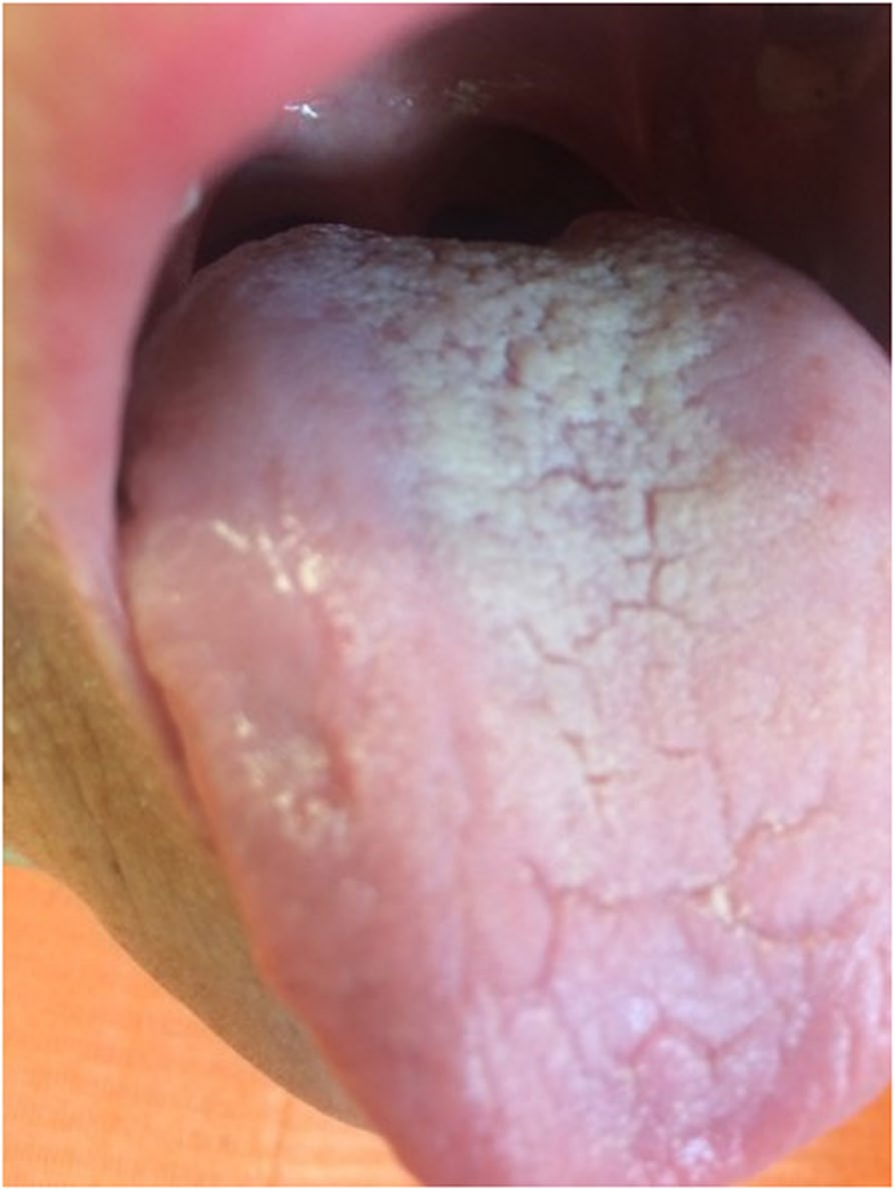
Candidiasis of the tongue in a 42-year-old man with chronic hepatitis C. Copyright Joanna Gałuszka-Garnuszek

**Fig. 4:**
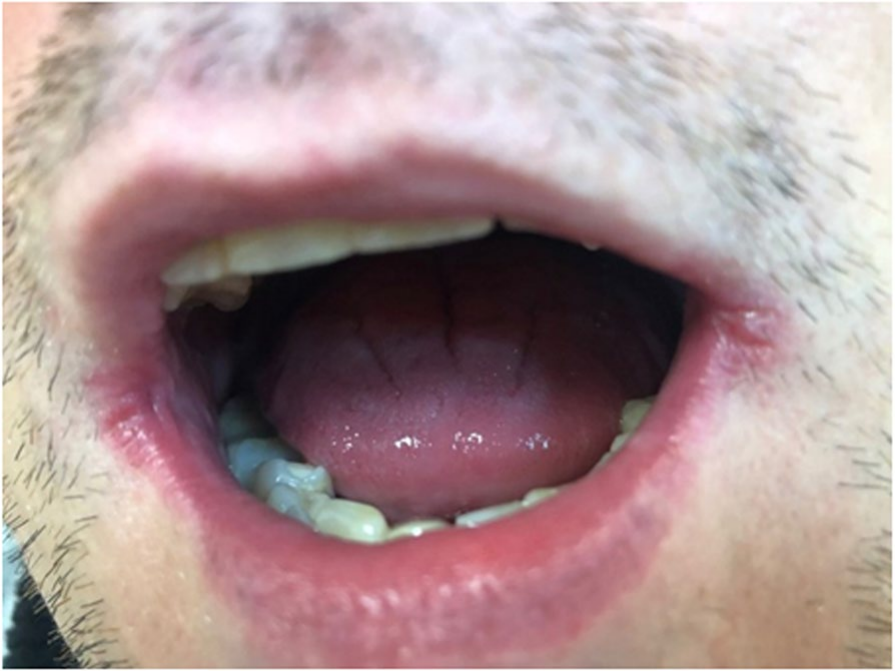
Angular cheilitis in a 47-year-old man with chronic hepatitis C. Copyright Joanna Gałuszka-Garnuszek

**Fig. 5:**
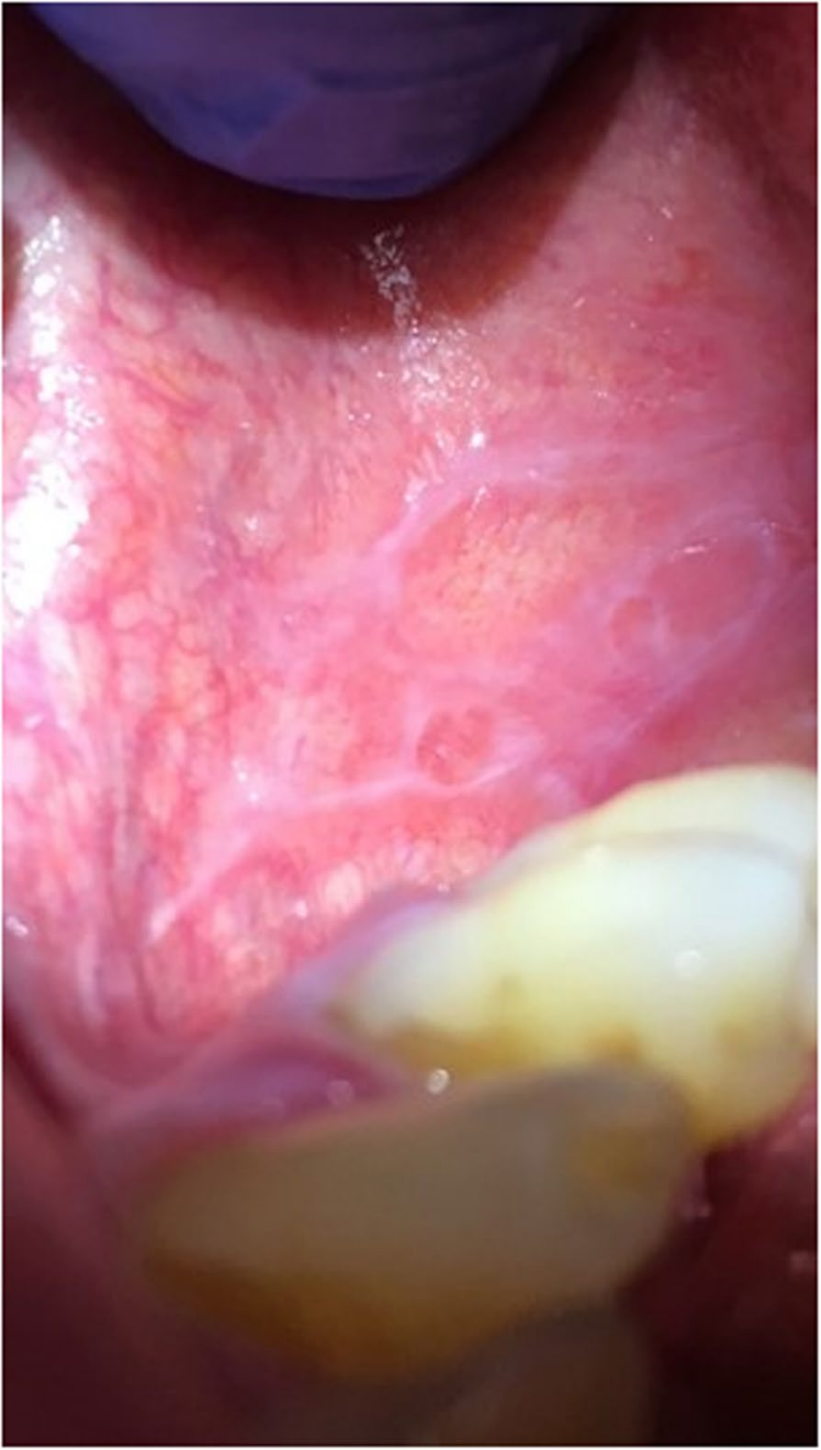
Wilson's Lichen Planus—reticular form on the buccal mucosa in an 81-year-old man with chronic hepatitis C. Copyright Joanna Gałuszka-Garnuszek

**Fig. 6:**
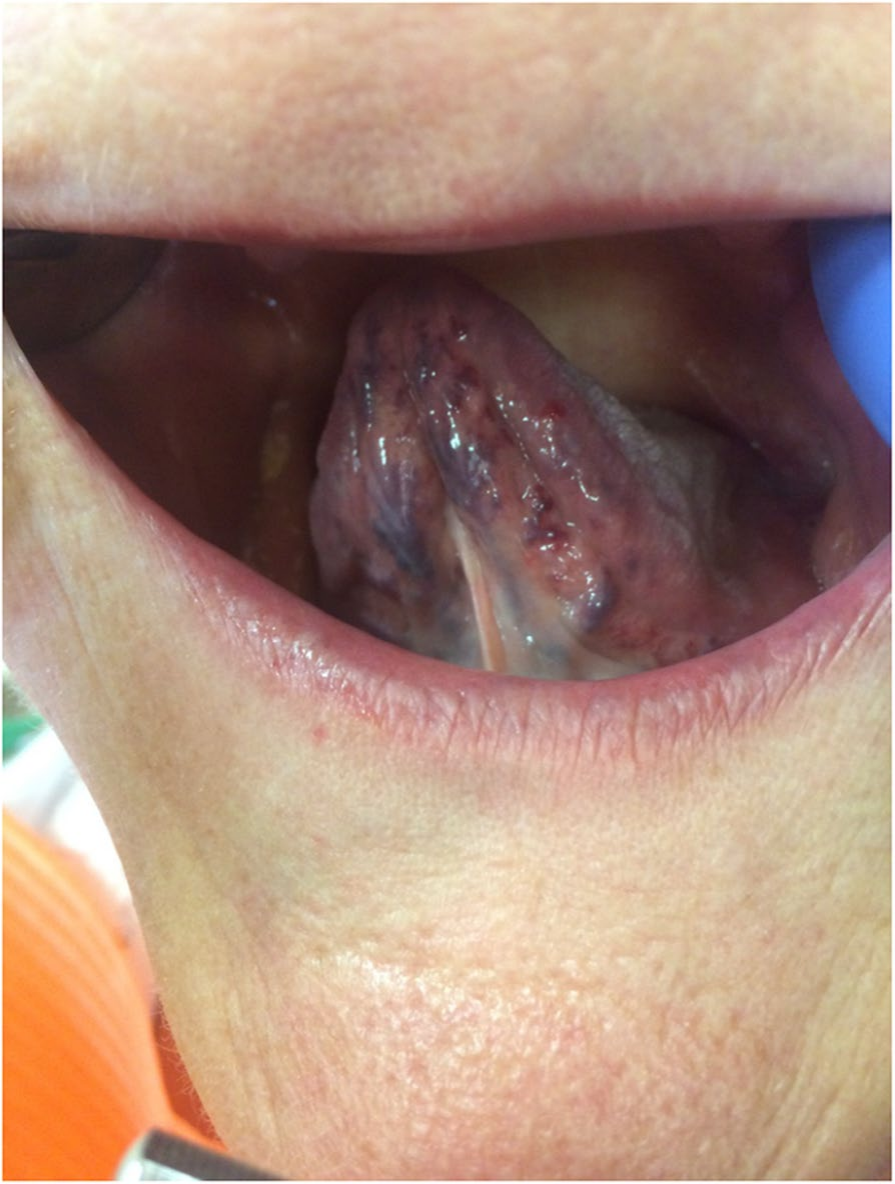
Sublingual varices in a 71-year-old female with chronic hepatitis C. Copyright Joanna Gałuszka-Garnuszek

Pathological changes were reported significantly more frequently in the study group, identified in 73.2% of individuals, compared to 32% in the control group (*p *< 0.0001) (Table 3). The most common pathology was oral candidiasis (Fig. 3), observed in 45.5% of patients with CHC, followed by angular cheilitis (Fig. 4). These conditions appeared significantly more often in the study group compared to the control group (*p* = 0.0169, *p* < 0.0001, *p* = 0.0003, respectively). Lichen planus (Fig. 5) was the third most common pathology, with 29 cases diagnosed in the CHC group, of which 9 cases with not obvius clinical diagnosis, were confirmed histopathologically. However, no statistically significant differences were noted between the study and control groups regarding the occurrence of the following pathologies: Mikulicz’s aphthae, papilloma, fibroma, and sublingual varices. Sublingual varices were present only in the study group, in 8 patients, three of whom had cirrhosis (Fig. 6). Interestingly, 7 out of these 8 patients were women.

**Table 3:** Pathological changes the oral mucosa in the study and control groups

Pathological changes of the oral mucosa, n(%)	Study population, *n* = 153	Control group, *n* = 50	p
Any pathological change	112 (73.2)	16 (32)	< 0.0001
Lichen planus	29 (19.0)	0	0.0003
Mikulicz’s aphthae	18 (11.8)	6 (12.0)	0.9643
Angular cheilitis	51 (33.3)	2 (4.0)	< 0.0001
Papilloma	7 (4.6)	0	0.1972
Fibroma	4 (2.6)	0	0.5739
Candidiasis	69 (45.1)	13 (26.0)	0.0169
Sublingual varices	8 (5.2)	0	0.2038

### The condition of the teeth, periodontium, and oral hygiene

The DMF showed similar values between the groups, with a median of 18 (11–26) in the study group and 17 (11–20) in the control group (*p* = 0.1778) (Table 1). The study group exhibited a significantly higher median number of teeth affected by caries and teeth lost due to caries compared to the control group (*p* = 0.0003 and *p* = 0.0232, respectively). Conversely, the study group had fewer teeth with dental fillings, with a median of 7 (1–10) versus 10 (8–13) in the control group (*p* < 0.0001). Oral hygiene, measured by the OHI-S, appeared poorer in the study group, with a median of 1.8 (1.15–2.95) compared to 1.1 (0–1.6) in the control group (*p* < 0.0001). Finally, patients with HCV infection required periodontal treatment more than twice as often as those in the control group (*p* < 0.0001).

### Characteristics of patients with HCV infection

All patients were evaluated before planned DAA therapy,. All had HCV RNA present at the time of assessment, the predominant genotype was GT1b documented in 127 individuals (83%) (Table 4). In most patients (64.1%), liver fibrosis was assessed as F1, while cirrhosis affected approximately 10% of patients. More than half of the patients exhibited at least one extrahepatic manifestation associated with HCV infection other than oral mucosal involvement. The most common extrahepatic manifestation was cryoglobulinemia, followed by autoimmune thyroid disease and thrombocytopenia. Over 90% of patients were treatment-naive for HCV, while the remaining had primarily experienced unsuccessful therapy, including eight patients treated with PegIFN + RBV, one patient treated with SOF + PegIFN + RBV, and one patient treated with SOF + RBV. The average time from their previous treatment to 2018 was 7.9 years, with a standard deviation of 3.8 years. The time since treatment ranged from a minimum of 3 years to a maximum of 14 years. Additionally, nearly three-quarters of patients presented with comorbidities, resulting in a high percentage requiring medication.

**Table 4:** Characteristics of patients with HCV infection

Parameter	Study population, *n* = 153
HCV RNA [mln IU/ml], median (Q1-Q3)	813,000.0 (343,000.0–2390000.0)
ALT [IU/l], median (Q1-Q3)	
Females	40 (29–61.5)
Males	59 (39–103)
Genotype, n (%)	
1	3 (2.0)
1a	3 (2.0)
1b	127 (83.0)
3	14 (9.1)
4	6 (3.9)
Liver Fibrosis (METAVIR), n (%)	
0	9 (5.9)
1	100 (65.4)
2	17 (11.1)
3	11 (7.2)
4	16 (10.4)
History of previous therapy	
Treatment-naïve	143 (93.5)
Treatment-experienced^a^	10 (6.5)
Extrahepatic manifestations of HCV infection unrelated to oral mucosa, n(%)	
Any manifestation	83 (54.9)
Cryoglobulinemia	70 (45.6)
Thrombocytopenia	5 (3.3)
Autoimmune Thyroid Diseases	12 (7.8)
Comorbidities, n (%)	
Any Disease	113 (73.9)
Hypertension	49 (32.0)
Diabetes	11 (7.2)
Autoimmune disease	11 (7.2)
Renal disease	10 (6.5)
Coronary artery disease	10 (6.5)
Non-HCC tumors	7 (4.6)
Concomitant medications, n (%)	92 (60.1)

*ALT* Alanine transaminase, *HCV* Hepatitis C virus, *Non-HCC* Non-hepatocellular carcinoma, *RNA* Ribonucleic acid

^a^Eight patients were treated with PegIFN + RBV, one with SOF + PegIFN + RBV, and one with SOF + RBV

### Factors influencing the occurrence of pathologies in the oral cavity

**Fig. 7:**
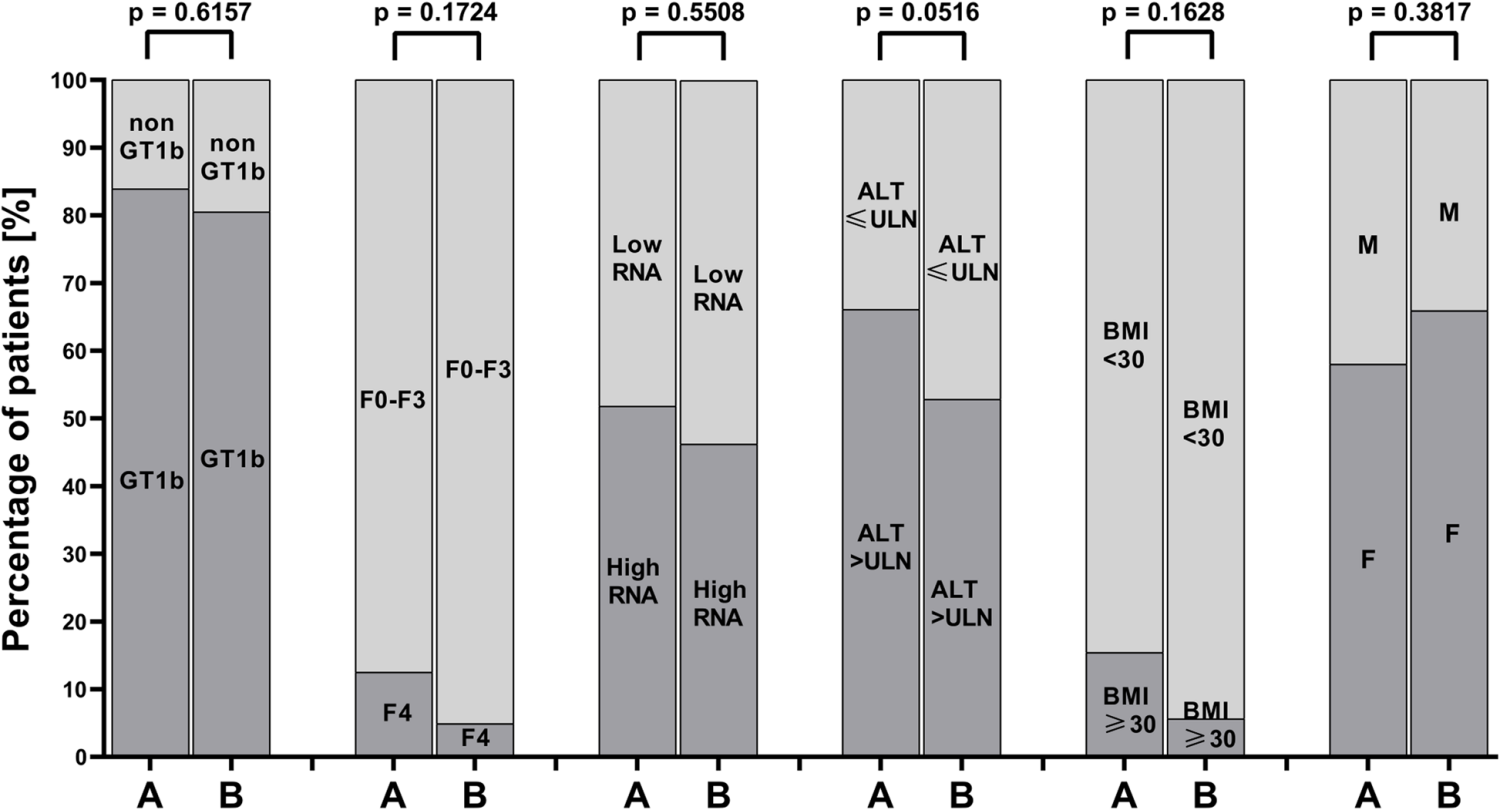
Comparison of patients with HCV infection with and without pathological changes based on genotype, degree of liver fibrosis, HCV RNA levels, ALT, BMI, and gender. **A** Patients with pathological changes. **B** Patients without pathological changes. Low HCV RNA—< 800,000 IU/mL. High HCV RNA- ≥ 800,000 IU/mL. ULN for females- 35 IU/L. ULN for males- 50 IU/L. ALT: Alanine aminotransferase; BMI: Body mass index; F: Female; F0-F4: Degree of liver fibrosis; GT: Genotype; M: Male; RNA: Ribonucleic acid; ULN: Upper limit of normal

**Fig. 8:**
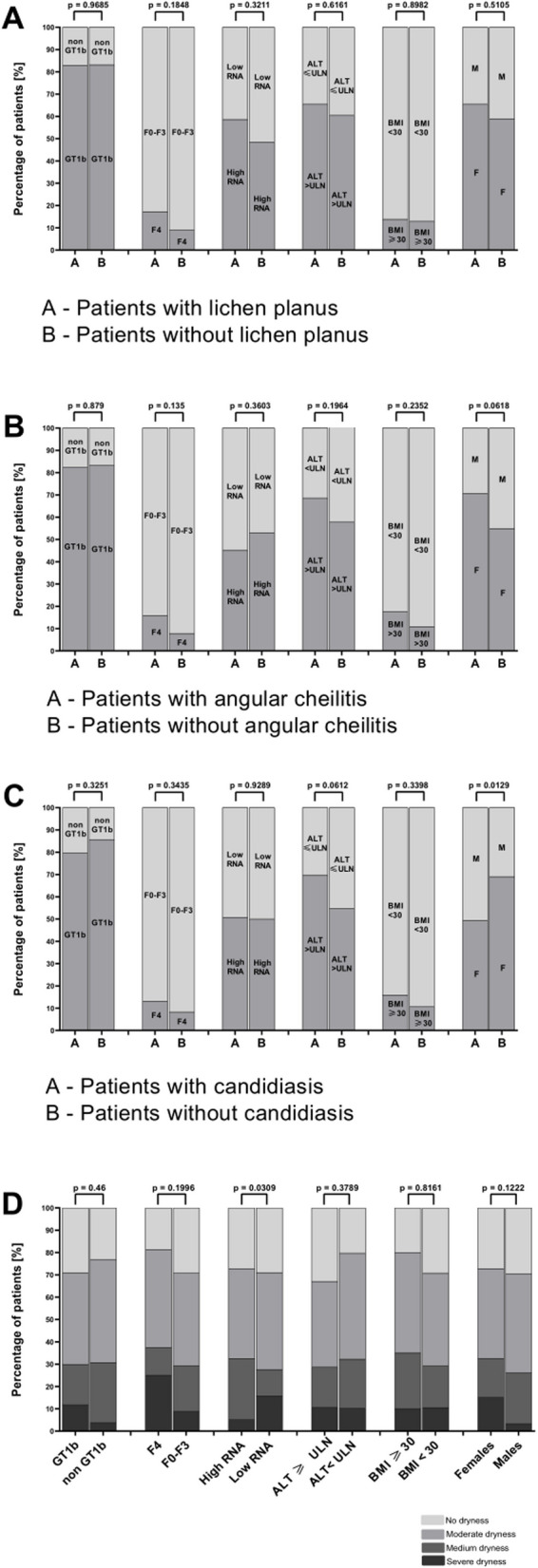
Comparison of patients with HCV infection with and without lichen planus (**A**), with and without angular cheilitis (**B**), with and without candidiasis (**C**) and mouth dryness severity according to the Challacombe scale based on genotype, degree of liver fibrosis, HCV RNA levels, ALT, BMI, and gender. Low HCV RNA—< 800,000 IU/mL. High HCV RNA- ≥ 800,000 IU/mL. ULN for females- 35 IU/L. ULN for males- 50 IU/L. ALT: Alanine aminotransferase; BMI: Boddy mass index; F: Female; F0-F4: Degree of liver fibrosis; GT1b: Genotype 1b; M: Male; non-GT1b: Non-Genotype 1b; RNA: Ribonucleic acid; ULN: Upper limit of normal

No significant relationships were found between HCV genotype, liver fibrosis stage, HCV viral load, ALT activity, BMI, or gender and the occurrence of oral mucosal pathologies, including oral lichen planus and angular cheilitis (Figs. 7, 8). However, a significant association (*p* = 0.0129) was documented between the male gender and the presence of oral candidiasis in the study group. (Fig. 8). Additionally, a statistically significant difference in mouth dryness among patients was observed depending on HCV RNA levels (*p* = 0.0309) (Fig. 8).

Independent positive predictors of the pathological changes within the oral cavity in the logistic regression analysis were age ≥ 40 years [OR = 4.343], GT1 infection (OR = 4.216), while the independent negative predictor was no mouth dryness (OR = 0.398). No association was documented for gender, BMI and oral hygiene (Table 5).

**Table 5:** Factors associated with the presence of pathologic within the oral cavity in logistic regression model in HCV-infected population

	**Effect measure**	**Wald stat**	**OR**	**95% Cl**	***P*-value**
Intercept		0.023	0.905	0.248–3.304	0.880
No mouth dryness	Yes	3.860	0.398	0.159–0.998	0.049
Insufficient oral hygiene	Yes	1.298	0.505	0.156–1.636	0.255
Age	≥ 40 years	7.681	4.343	1.537–12.272	0.006
Gender	Males	2.087	0.496	0.191–1.284	0.149
GT1b	Yes	4.911	4.216	1.181–15.052	0.027
BMI	≥ 30	2.900	6.727	0.750–60.347	0.089

*BMI* Body mass index, *CI* Confidence interval, *GT* Genotype, *OR* Odds ratio

## Discussion

Up to two-thirds of HCV-infected patients experience a wide range of extrahepatic symptoms with differences depending on the geographic region and population studied [6, 21, 22]. These extrahepatic conditions are particularly relevant to dental professionals, as they may serve as early, non-invasive indicators of infection in otherwise asymptomatic individuals. This highlights an important opportunity for dental practitioners to play a proactive role in identifying potential HCV infections and referring patients for appropriate diagnostic testing and antiviral therapy. Effective treatment of such patients with direct-acting antiviral drugs at an early stage of the disease, before symptoms of liver disease and serious complications, including cirrhosis and HCC, appear. Although oral abnormalities can provide conspicuous clues about the possible infection through non-invasive examination, they remain understudied with researchers mostly focusing on the most common conditions—oral lichen planus (OLP), xerostomia, and oral cavity cancer [23, 24]. In our real-world study, we focused on collecting and analyzing data from over 150 patients to determine the prevalence of various oral symptoms and create a comprehensive profile of HCV-infected patients who experienced them.

One of the relevant directions in recent research concerns the impact of DAA therapy on extrahepatic manifestations. Di Stasio et al. demonstrated that DAA treatment may lead to clinical improvement of OLP in HCV-infected patients [25]. While these findings are of high clinical importance, we would like to note that all patients in our study were assessed prior to the initiation of DAA therapy. As such, the effects of antiviral treatment on oral lesions were not within the scope of our analysis.

We documented that mucosal lesions occurred more than twice as often as in the control group Similar prevalence was reported in Brazil where 68.4% of all CHC patients had at least one oral mucosal condition, however, the study didn’t include any control group [26]. The most commonly reported oral abnormality we identified was candidiasis, followed by angular cheilitis and OLP. In the observational retrospective study carried out in Egypt involving fifty patients, OLP was the most common pathological change (20% vs. 0% in the control group), while xerostomia was mentioned as the most prominent subjective oral symptom (40% vs. 12% in the controls) which is consistent with our results (47.1% vs. 6%) [27].

In our analysis, a logistic regression model identified age over 40 as the strongest factor independently increasing the risk of developing oral abnormalities. A similar observation was found in the Taiwanian study in which individuals aged 40–49 years had the strongest positive association with an increased risk of oral cavity cancer [28]. We documented also increased odds of developing pathologies within the oral cavity was infection with GT1b, while lack of xerostomia decreased the risk. It is a well-known fact that inappropriate salivary flow can contribute to other oral pathologies such as dental caries, taste disturbances, and candidiasis [29].

The pathogenetic link between OLP and CHC has been well documented in the scientific literature, with the first reports dating back to 1991 [30]. According to some researchers, it is the most common oral manifestation in HCV-infected patients, in our study, OLP appeared as the third most common oral pathology [27]. A meta-analysis by Lodi et al. covering six studies and over two thousand patients, confirmed a positive correlation between the presence of OLP and HCV infection [31]. Although some reports suggest a higher incidence of OLP among individuals with HCV GT1, our findings align with the prevailing hypothesis that viral factors, such as GT or viral load, are not significantly correlated with the occurrence of OLP [23]. Additionally, despite some authors reporting a positive correlation between ALT activity and the OLP, our study did not confirm this association [32]. We also did not demonstrate a relationship with factors such as gender, BMI and the stage of liver fibrosis. Notable, although OLP can occur, exacerbate, and persist during IFN therapy for CHC, in our study, OLP was diagnosed in only one patient with a history of IFN-based treatment [33].

We have described not only manifestations whose association with HCV is well known and documented but also such conditions that, although not directly related to infection, were more common in patients with CHC, including oral candidiasis. It was the pathological mucosal lesion most frequently reported in the study population, regardless of the presence of HCV infection but was significantly more common in the HCV group. It is important to note that while the pathogenetic link between HCV infection and OLP is undisputed, in the case of candidiasis, we are more concerned with the co-occurrence of HCV and this condition. Dentists should consider HCV evaluation in patients with recurrent or treatment-resistant candidiasis, especially in the absence of common local risk factors. According to other studies, the frequency may vary depending on the population and geographic region analyzed. In a case–control study conducted in Egypt, oral candidiasis occurred in only 5.1% of HCV-infected patients and was more common among healthy controls (6.7%) [34]. A Brazilian cross-sectional study reported 39 cases of oral candidiasis in patients with CHC (18.1%), but no control group was included for comparison [26]. In our study, there was no statistically significant association between HCV genotype, viral load, HBV co-infection, BMI or ALT levels, and candidiasis; the only parameter for which we documented an association with candidiasis was male gender.

In a case–control study conducted in over one thousand five hundred patients, authors identified independent predictors of candidiasis such as hyposalivation, presence of Sjogren's syndrome, and history of autoimmune disorders, while no significant link was found for age, race, and cigarette smoking. Not surprisingly, diabetes mellitus was an independent predictor of Candida overgrowth among patients already suffering from oral dryness [35].

We documented that the second most common pathological change in the oral cavity after candidiasis, present significantly more often in HCV-infected patients than in the control group, was angular cheilitis. Both conditions are not pathogenetically related to HCV but rather result from altered immunology in infected patients. Additionally, both conditions have similar risk factors, so it is not surprising that almost half of HCV patients with candidiasis also had angular cheilitis [36]. No significant difference regarding genotype, degree of liver fibrosis, HCV RNA levels, ALT, BMI, and gender was found between groups divided based on the presence of angular cheilitis. Analyzing the oral mucosa changes in CHC patients, Sulka et al. found that only one cirrhotic patient out of 16 had candidiasis, while angular cheilitis wasn’t reported at all [37]. In another study, carried out among patients undergoing IFN therapy, angular cheilitis were found in 21.43% of patients with oral mucosal lesions, all women [38].

A possible pathological link has been proposed between HCV infection and the development of periodontal disease based on two main pathways – insulin resistance and chronic inflammatory process in the liver. Diabetes is a well-known risk factor for periodontal disease, and patients with CHC are more prone to developing insulin resistance and diabetes due to multiple mechanisms such as metabolic syndrome, liver steatosis, and chronic inflammatory state in the liver. Moreover, impaired hepatic function due to HCV infection can impact nonspecific immune activity leading to chronic inflammation which can further insulin resistance, and impede the defense against periodontal pathogenic microorganisms [39, 40]. When assessing our study sample for CPITN we observed a significantly higher number of patients with calculus and shallow pockets in need of comprehensive treatment among the HCV-infected group compared to healthy controls. In a cross-sectional study, data from over five thousand patients aged 20 or older was obtained from the NHANES database [41]. Multivariate logistic regression analysis revealed a significant association between HCV infection and periodontitis. Moreover, the analysis revealed that patients with moderate and severe periodontitis had increased odds of CHC compared to healthy individuals.

An interesting observation in our study was the documentation of sublingual varices not only in patients with liver cirrhosis, which may suggest their multifactorial etiology. Notably, 7 of these eight patients were women, further indicating potential gender-related factors, that require more extensive analysis into this phenomenon could inform screening guidelines for both gastroenterologists and dental professionals.

In the studied population, patients infected with HCV reported subjective oral symptoms twice as often as compared to the control group, and the most common of them was dry mouth. Although xerostomia in HCV-infected individuals may resemble the symptoms observed in primary Sjögren's syndrome, the underlying mechanisms are probably different, evidence indicating the presence of HCV RNA in the salivary glands supports the hypothesis of a link between xerostomia and HCV [24, 42]. Although our study did not include an objective diagnosis of Sjögren's syndrome, we documented a significantly higher incidence of dry mouth compared to controls. This finding is consistent with other studies indicating that between 20 and 80% of HCV-infected patients may have salivary or tear secretion disorders [43, 44]. A meta-analysis of ten studies confirmed the relationship between dry mouth and HCV infection, estimating the average risk level at 3.3%, which is six times higher than in uninfected people, a result consistent with our findings [45]. On the other hand, other research found no evidence of HCV infection among patients with well-defined primary SS, which emphasizes the uncertainty in this relationship [46]. Nonetheless, the association between HCV and reduced salivary function supports the notion that dentists should inquire about dry mouth symptoms as part of routine exams.

In our analysis, pain and burning in the mouth also occurred statistically more often in patients with CHC compared to the control group. Current literature does not propose a direct relationship between this pathology and HCV infection; some researchers speculate that the secondary form of burning mouth syndrome may be related to xerostomia and fungal superinfections [47, 48]. Their presence, particularly in middle-aged or elderly patients, should not be dismissed as idiopathic without systemic workup.

Noteworthy, no one with HCV infection in our study was diagnosed with oral cancer, which may be due to the small size of the cohort, as an analysis from Taiwan involving 21,000 patients showed a 2.28-fold higher risk of oral cancer compared to uninfected individuals, emphasizing the importance of routine oral cancer screening in this population [28].

The relatively small sample size, which may affect the generalizability of the results to a broader population of HCV-infected patients, is one of the limitations of our study that must be acknowledged. Additionally, the control group may not be fully representative of the general population, which may introduce selection bias. Furthermore, the subjective assessment of verbal complaints and the use of self-reported measures such as the NRS for pain are inherently subject to individual interpretation, which may impact the accuracy of data collected. It is also important to bear in mind the potential for respondents to be concessive to agreeing with binary response options, which may have caused variations in data collection. Clinical examination and diagnosis of oral mucosa diseases have relied primarily on visual assessment and patient interviews which are inherently prone to possible errors in reminiscing past symptoms leading to potential recall bias, may not provide the sensitivity and specificity of more advanced diagnostic techniques. Although the diagnosis of OLP was based on characteristic clinical presentation, histopathological confirmation was performed only in uncertain cases (9 out of 29), which may introduce diagnostic variability. Another limitation of this study is the lack of detailed data on smoking and alcohol consumption history. While none of the participants were smokers or alcohol-dependent at the time of therapy initiation, and all declared they had never smoked or consumed alcohol in the past, these declarations do not provide full certainty regarding their previous habits, which could potentially affect the development of oral pathologies.

The study did not take into account potential confounding factors such as history of smoking and dietary habits, which are known to affect oral health and may influence the results. Finally, data were collected from a single treatment center, which limits the generalizability of the results to other settings and populations. However, this limitation is also a strength of the analysis because the patients were examined by the same physician according to a standardized protocol. To further improve the external validity of these findings, future research should include multicenter studies that can control for confounders and account for greater diversity in patient populations.

## Conclusions

In patients chronically infected with HCV, oral mucosal pathologies were significantly more prevalent compared to controls. The most frequently observed conditions included oral mucosal candidiasis, angular cheilitis, and OLP. Subjective symptoms reported most frequently were dry mouth, oral pain, and burning sensation. Age ≥ 40 years and GT1b HCV proved to be independent positive predictors of the presence of oral mucosal lesions.

## Data Availability

The datasets used and/or analysed during the current study are available from the corresponding author on reasonable request.
